# Monitoring the occurrence of diabetes mellitus and its major complications: the combined use of different administrative databases

**DOI:** 10.1186/1475-2840-6-5

**Published:** 2007-02-15

**Authors:** Stefano Brocco, Cristiana Visentin, Ugo Fedeli, Elena Schievano, Angelo Avogaro, Margherita Andretta, Francesco Avossa, Paolo Spolaore

**Affiliations:** 1 Epidemiological Department, Veneto Region, Italy; 2 Department of clinical and experimental medicine, University of Padua, Italy; 3 Pharmaceutical Department, Veneto Region, Italy

## Abstract

**Objective:**

Diabetes mellitus is a growing public health problem, for which efficient and timely surveillance is a key policy. Administrative databases offer relevant opportunities for this purpose. We aim to monitor the incidence of diabetes and its major complications using administrative data.

**Study design and methods:**

We study a population of about 850000 inhabitants in the Veneto Region (Italy) from the end of year 2001 to the end of year 2004. We use four administrative databases with record linkage. Databases of drug prescriptions and of exemptions from medical charge were linked to identify diabetic subjects; hospital discharge records and mortality data were used for the assessment of macrovascular and renal complications and vital status.

**Results:**

We identified 30230 and 34620 diabetic subjects at the start and at the end of the study respectively. The row prevalence increased from 38.3/1000 (95% CI 37.2 – 39.5) to 43.2/1000 (95% CI 42.3 – 44) for males and from 34.7/1000 (95% CI 33.9 – 35.5) to 38.1/1000 (95% CI 37.4 – 39) for females. The mean row incidence is 5.3/1000 (95% CI 5 – 5.6) person years for males and 4.8/1000 (95% CI 4.4 – 5.2) person years for females. The rate of hospitalisations for cardiovascular or kidney diseases is greatly increased in diabetic people with respect to non diabetics for both genders. The mortality relative risk is particularly important in younger age classes: diabetic males and females aged 45–64 years present relative risk for death of 1.7 (95% CI 1.58 – 1.88) and 2.6 (95% CI 2.29 – 2.97) respectively.

**Conclusion:**

This study provides a feasible and efficient method to determine and monitor the incidence and prevalence of diabetes and the occurrence of its complications along with indexes of morbidity and mortality.

## Background

For an adequate health planning, policy-makers need continuous, accurate and timely data about the main diseases affecting the population with a great impact on utilization of health services. Diabetes mellitus represents an important and growing problem in developed countries for its prevalence and the high risk of major complications [1,2]. The burden of this disease is increasing worldwide both for the progressive ageing of population and for the worsening of life-style. This increase is expected both for type 2 and for type 1 diabetes [3,4].

A growing body of scientific literature is now available on the use of administrative data for surveillance purposes. Surveillance with administrative data is feasible, timely, little expensive and involves, with a wide coverage and continuity, a high number of subjects. For these feature, administrative databases are widely used for epidemiologic purposes and health services research.

Diabetic disease is an ideal model for surveillance with administrative data. First, monitoring diabetes and its complications is a key policy in public health surveillance for defining the burden of disease, for planning health services, for evaluating strategies in disease prevention and control and assessing outcomes. Second, diabetes presents a feasible detection and follow-up by the use of current data, i.e, a highly specific pharmacologic treatment, high rate of hospitalisation and, at least in Italy, dedicated diabetes care units when the disease is certified; moreover, since that diabetic subjects have some facilities for out of hospital care, local health authorities keep a register of subjects with a diagnosis of diabetes (as well as other chronic diseases) for administrative purposes. Various approaches have been proposed to assess the burden of diabetes and its consequences with administrative data [5-10], and their validity has been widely demonstrated [11,12] and recently reviewed [13]. However, the rare opportunity given by the Italian health system of two different administrative databases with a high traceability of diabetic subjects has not been sufficiently explored; furthermore the studies about the epidemiology of diabetes and its complications with administrative data are limited to particular aspects (incidence or prevalence or complications or quality of care), without a global picture of the diabetic disease.

Therefore the aim of this study was to propose a model for the surveillance of diabetes and its complications with the use of four different archives of administrative data (hospital discharge records, mortality data, drug prescriptions and exemption from medical charge) with record-linkage between the four databases. Specifically we wish to determine the prevalence and the incidence of diabetes and the detection of its major complications (cardiovascular and renal) and mortality in diabetic patients.

## Methods

This is a retrospective cohort study, based on administrative data from 2001 to 2004.

We consider data from three local Health authorities in the Veneto Region (North-East Italy). The total population observed is about 850000 people (20% of the regional population).

### Data sources

We used hospital discharge records (HDRs, years 2002–2004), mortality data (MD, years 2002–2003), drug prescriptions (DP, years 2001–2004) and exemption of medical charge (EMC, years 2001–2004). We provide a brief description of the four databases, with more details about EMC, a peculiarity of the Italian Health System.

### Hospital discharge records

HDRs contain personal data (first name, last name, date and place of birth, residence, fiscal identification number and health service identification number) and hospitalisation data (date and ward of admission, date and ward of discharge, date and ward of transfers, if any). There are one principal discharge diagnosis and up to five other secondary diagnosis; there are up to six medical or surgical procedures. Since year 2000, diagnosis and procedures are codified according to ICD-9-CM codes (1997 version).

### Mortality data

Each local health Authority must keep and maintain a register of mortality data. The MD records contain personal data (first name, last name, date and place of birth, residence and fiscal number) and a unique cause of death, codified with ICD-9 codes (according to rules of national institute of statistics, adapted from World Health Organisation indications).

### Drug prescriptions

Archives of drug prescriptions are collected by local health authorities and contain data about the patient, about the prescribing physician and about the drugs. Drugs are classified according the Anatomical Therapeutic Chemical (ATC) classification system. We extract the records with the following ACT codes :A10A (insulin) and A10B (oral antidiabetic drugs).

### Exemption from medical charge

In the Veneto Region, public hospital care is free of charge. Subjects aged under 65 years must pay a part of out-of-hospital care (laboratory tests, specialistic visits,...) and must contribute to drug costs. If a subject is certified as diabetic, all the out-of-hospital care concerning diabetes is free of charge. This comprises laboratory controls (HbA1C, lipids, microalbuminuria...) periodical clinical evaluations (diabetologist, ophthalmologist...), instrumental examinations (ECG, ecocolordoppler...,), self-monitoring glucose strips and antidiabetic drugs (and needles for insulin, when needed). Thus, the local health authorities keep a register of the subjects certified as diabetics. In a recent survey in the Veneto Region we established that the positive predictive value of EMC for the diagnosis of diabetes is 98% in a sample of people aged 18–65 years (unpublished data).

### Record-linkage

The four databases employed different personal identification data; therefore, we consider as valid only those records that match with the regional archive of people covered by health assistance (i.e., all people who is regularly resident in the Veneto Region). Even if with different identification data, HDRs, mortality data and EMCs are all connected through the archive of people covered by health assistance; linkage of these archives therefore approximates 100%. Drug prescriptions are linked with the archive of people covered by health assistance by a personal identification code: about 5% of single drug prescriptions did not link. Since that diabetic people usually have more than one prescription the probability that a diabetic subject with multiple drug prescriptions is missed by our system is quite low.

People coming from developing countries presents some problems in the linkage, because of the inaccuracy or the lack of personal data. Since that in the Veneto Region they account for 5% of the total population and since that their age is young, we are confident that the potential under detection of diabetics is of little extent.

### Prevalence and incidence

Prevalent cases are defined at the end of each year. For example, we compute prevalence of diabetes at the end of year 2001 considering in the numerator all subject with one or more prescriptions of antidiabetic drugs in the last six months of the years 2001 and/or subjects present in the EMC at the end of 2001. For subjects present only in DP database two or more prescriptions of antidiabetic drugs are required for the labelling as diabetic people. Women aged 15–44 years (reproductive age) with only one prescription of insulin or traceable in the EMC database only temporarily, were excluded to avoid the inclusion of gestational diabetes.

Incident cases of diabetes are identified each year by subtracting from diabetic subjects at the end of the year those already identified at the end of the previous year: subjects defined as diabetic at the end of the years 2002 but not present in the PD or EMC databases at the end of 2001 were considered incident cases in the year 2002.

Data are presented at the start and at the end of the study period as row and standardized prevalence and incidence rates as well as age and gender specific prevalence and incidence rates.

### Hospitalised complications and mortality

For the entire period of the study, we calculated the hospitalisation rates among diabetics and non diabetics, through the record linkage of the previously identified diabetic population with the database of HDRs.

We assess the hospitalisation for the following nine causes: all causes, ischemic heart disease, acute myocardial infarction, heart failure, stroke, all cerebrovascular diseases (comprising stroke), peripheral vascular diseases, low limb amputations, renal diseases. In table 1 are listed the ICD-9-CM codes utilised for the identification of these conditions.

**Table 1:** ICD-9-CM codes for the identification of complications in Hospital Discharge Records

Condition	ICD-9-CM codes	Fields
Ischemic heart disease	410xx, 411xx, 412, 413x, 414xx	Primary diagnosis
Acute myocardial infarction	410x1	All diagnostic fields
Heart failure	428x, 7855x	Primary diagnosis
Cerebrovascular diseases	430, 431, 432x, 433xx, 434xx, 435, 436, 437x, 438	Primary diagnosis
	3811, 3812, 3831, 3832, 3841, 3842	All procedural fields
Stroke	430, 431, 433x1, 434x1, 436	Primary diagnosis
Peripheral vascular disease	2507x, 440xx, 442xx, 443xx, 444xx, 447x, 459xx, 7854	Primary diagnosis
	V434, 3813, 3814, 3815, 3816, 3818, 3925, 3929	All procedural fields
Low limb amputations	895x, 896x, 897x	All diagnostic fields
	8410, 8411, 8412, 8413, 8414, 8415, 8416, 8417, 8418, 8419, 8622	All procedural fields
Kidney diseases	2504x, 403xx, 404xx, 581xx, 584x, 585, 586, 588x	Primary diagnosis
	3895, 3927, 3942, 3995, 5493	All procedural fields

For each class of age and gender we report the rate of hospitalisation for each cause in diabetics and the relative risk with respect to non diabetic people.

The record-linkage between the diabetic population and the archive of mortality data (years 2002–2003) allows to compute mortality rates in diabetics and non diabetics. Because of the known low accuracy of the causes of death in death certificates, and the lack of multiple causes and concomitant diseases in our database, we did not use death certificates for the analysis of the causes of death. Even mortality data are presented as gender and age specific rates and relative risk.

### Statistical analysis

For row age and gender specific prevalence and incidence rates, 95% confidence intervals are determined with the approximation to the normal distribution, for relative risks for hospitalisation and mortality we assumed a log-normal distribution. Standard errors are computed with the usual methods[14].

The standardization for prevalence and incidence rates was performed with the direct method, using age and gender specific rates for the following classes of age: 0–4, 5–14, 15–24, 25–34, 35–44, 45–54, 55–64, 65–74, 75–84, 85 and over. The standard population is the Italian population of the year 2000, source national institute of statistic)

Record linkage and analysis was performed with the statistical software package SAS vers 9.

## Results

### Occurrence of diabetes

The diabetic subjects identified at the start and at the end of the study are 30230 and 34620 respectively. There is a slight prevalence of male gender (52%). Of importance, diabetic subjects are identified by the presence in the EMC database only in 16% of cases, by the presence in the DP database in the 24% of cases, and by the presence in both databases in 60% of cases. The proportion of diabetic patients treated only with oral antidiabetic drugs is 65%, while 19% of patients assumes either insulin only (60%) or in combination with oral antidiabetic drugs (40%).

The row prevalence for males is 38.3/1000 in the beginning and 43.2/1000 at the end of the study ; for females 34.7/1000 and 38.1/1000, respectively (p < 0.001). The standardised prevalence rates in the beginning and at the end of the study are 39/1000 and 43.5/1000 for males, 34.7/1000 and 37.6/1000 for females, respectively (p < 0.001). Row and standardised prevalence rates both increase by about 10% through the three years studied.

The row incidence rate of diabetes for the period 2002 – 2004 is 5.3/1000 person years for males and 4.8/1000 person years for females. The standardized incidence rate of diabetes for the same period is 5.5/1000 person years for males and 5/1000 person years for females.

**Figure 1:**
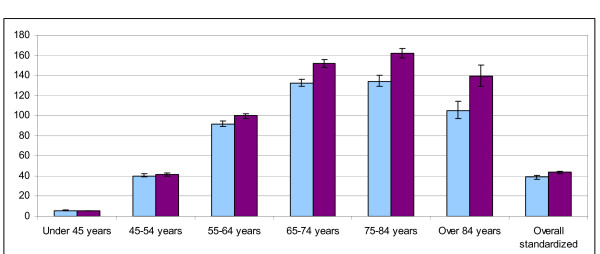
Prevalence (*1000 subjects) of diabetes in males.

**Figure 2:**
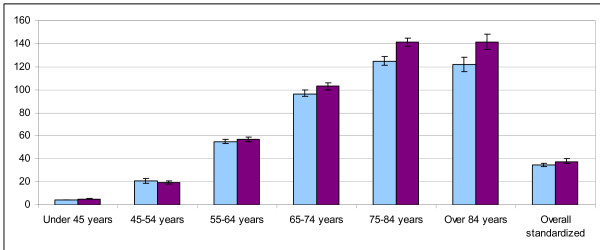
Prevalence (*1000 subjects) of diabetes in females.

The age and sex specific prevalence and incidence rates are shown in table 2 and in figures 1 and 2.

**Table 2:** Age and gender specific occurrences of diabetes.

	Prevalence (*1000 subjects)				Incidence (*1000 person years)	
	31/12/2001		31/12/2004		Mean 2002 – 2004	

	males	females	males	females	males	females

Under 45 years	5.6	4.2	5.5	5	1.3	1.1
	5.3–5.9	4–4.5	5.2–5.8	4.7–5.3	1.2–1.4	1–1.2
45–54 years	40.1	21	41.2	19.4	6.7	3.5
	39–42	19–23	40–43	18–21	6.3–7.2	3.2–3.8
55–64 years	91.2	55	99.9	56.6	12.8	7.6
	89–95	53–57	97–102	55–59	12.2–13.5	7.1–8.1
65–74 years	132	96.4	152	102.9	17	12.4
	129–136	94–100	148–156	100–106	16.1–18	11.7–13.1
75–84 years	134	125	162.1	141.4	15.9	15.5
	129–140	121–129	157–167	138–145	14.7–17.1	14.6–16.4
Over 84 years	105	122	139.3	141.6	16.2	15.5
	97–114	116–128	129–150	135–148	13.8–18.9	14–17
Overall standardized	39	34.7	43.5	37.6	5.5	5.0
	37-41	33-36	42-45	36-40	(5.2-5.8)	(4.7-5.3)

### Major complications and mortality

**Figure 3:**
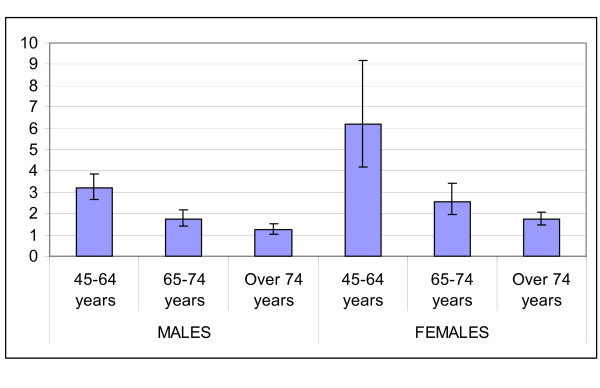
Relative Risk of hospitalisation for acute myocardial infarction.

**Figure 4:**
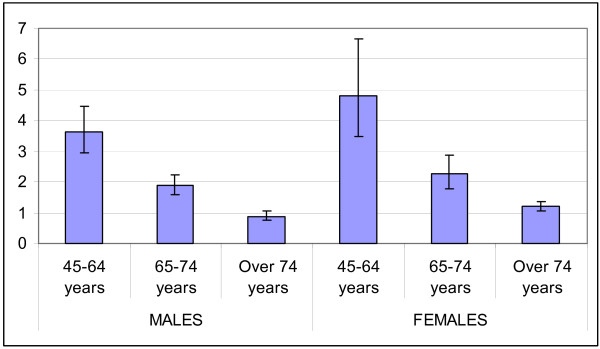
Relative Risk of hospitalisation for Stroke.

**Figure 5:**
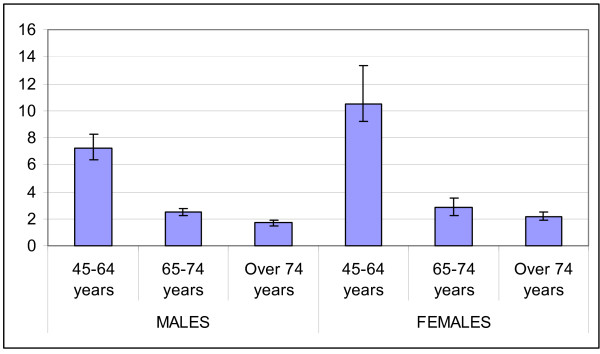
Relative Risk of hospitalisation for peripheral vascular diseases.

**Figure 6:**
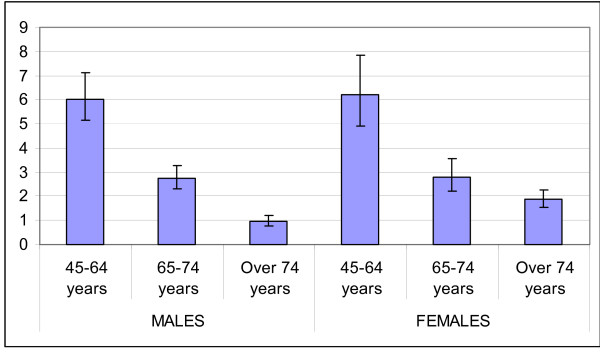
Relative Risk of hospitalisation for kidney diseases.

Diabetic patients show increased rates of hospitalisation for all causes and for cardiovascular and renal causes (table 3 and figures 3, 4, 5 and 6) as compared to non-diabetic subjects. The effect of diabetes in terms of relative risk is present in each group of age and for each cause, except for cerebrovascular diseases, stroke and kidney diseases for elderly males. Absolute rates are higher specifically in older males; on the contrary, relative risk is higher distinctively in younger women. For younger classes of age and for certain causes (heart failure, peripheral vascular diseases, kidney diseases) the risk of hospitalisation among diabetic subjects is even five-fold or more respect to non diabetic people.

**Table 3:** Hospitalisation* and mortality in diabetic subjects, by age and gender.

		45–64 years		65–74 years		Over 74 years	
		males	females	males	females	males	females

All hospitalisations	Rates	376	363	569	417	668	527
	RR	2.04	2.04	1.33	1.47	1.07	1.19
	95% ci	1.99–2.08	1.99–2.1	1.31–1.35	1.43–1.50	1.05–1.08	1.17–1.21
Ischemic heart disease	Rates	29.3	11.1	36.8	22.4	37.4	27.9
	RR	3.37	7.36	1.72	3.26	1.18	1.72
	95% ci	3.06–3.7	5.98–9.04	1.56–1.90	2.83–3.75	1.05–1.32	1.55–1.91
Acute myocardial infarction	Rates	8.1	3	8.9	5.1	13	11.1
	RR	3.22	6.21	1.76	2.58	1.23	1.74
	95% ci	2.69–3.85	4.2–9.19	1.43–2.17	1.94–3.45	1.01–1.5	1.48–2.06
Heart failure	Rates	9.2	4.1	21.9	13	39.3	34.7
	RR	6.14	9.28	2.55	3.15	1.23	1.42
	95% ci	5.11–7.36	6.52–13.19	2.22–2.93	2.62–3.79	1.09–1.38	1.29–1.55
Cerebrovascular diseases	Rates	13.2	9.2	32.6	19.1	37.3	35.4
	RR	3.64	4.61	2.03	2.34	0.95	1.28
	95% ci	3.16–4.19	3.71–5.72	1.82–2.26	2.02–2.71	0.58–1.06	1.17–1.40
Stroke	Rates	6.2	4.2	12.5	7.3	15.3	16.5
	RR	3.64	4.81	1.88	2.28	0.88	1.20
	95% ci	2.96–4.48	3.48–6.66	1.58–2.25	1.79–2.89	0.74–1.06	1.05–1.37
Peripheral vascular diseases	Rates	20.2	8.7	34.6	8.8	35.7	14.9
	RR	7.25	10.48	2.49	2.84	1.7	2.17
	95% ci	6.4–8.22	8.21–13.37	2.24–2.78	2.27–3.55	1.5–1.92	1.87–2.51
Low limb amputations	Rates	6.7	2.8	6.2	1.7	6	4.7
	RR	13.01	10.27	6.1	2.85	2.38	3.04
	95% ci	10.16–16.66	6.68–15.78	4.47–8.31	1.72–4.7	1.73–3.27	2.31–3.99
Kidney diseases	Rates	11.3	8.5	13.6	7.8	10.7	8
	RR	6.04	6.21	2.75	2.8	0.97	1.88
	95% ci	5.13–7.12	4.93–7.83	2.31–3.29	2.21–3.54	0.78–1.21	1.54–2.28
Mortality	Rates	9.23	6.93	26.4	16.1	83.6	63.4
	RR	1.72	2.62	1.1	1.86	0.98	1.02
	95% ci	1.58–1.88	2.29–2.97	1.02–1.21	1.74–1.98	0.91–1.06	0.94–1.11

* Rates (*1000) in diabetics and relative risk with 95% confidence interval (respect to non diabetics)

The relative risk of mortality in diabetic subjects aged 45–64 years is increased both for males and females (1.7 and 2.6 respectively), while there is no difference in the risk of death between diabetics and non diabetics in subjects older than 74 years.

## Discussion

This study provides a useful model to identify diabetic subjects, to describe the burden of the disease in terms of diabetic complications, health services utilisation, and health outcome. The combined use of drug prescription and of exemption from medical charges to identify diabetic patients allows a significant increase of sensitivity, maintaining a high specificity, with respect to use of drug prescription only. In effect, with the DP, diabetic subjects that not assume antidiabetic drugs cannot be traced, while they could be present in the archive of EMC. In our study 16% of the total diabetic population is detected by the presence in the EMC database only.

A questionable issue is the accuracy of the EMC database in the identification of diabetic people. We reported above in the paper a very high positive predictive value of this database for the identification of diabetic subjects. The sensitivity, however, may be suboptimal: Gnavi et al [15] reported that 73% of subjects assuming antidiabetic drugs are traceable in the database of exemption from medical charge, an amount similar to our findings (71%). Nevertheless, diabetic subjects not present in the EMC database can be traced with DP, so we are confident that the accuracy of the combined use of the two databases is characterised by high sensitivity and specificity.

The estimates of prevalence, incidence and time trends are consistent with other national [16,17] and international surveys [18,19], conducted with other more accurate but expensive methodologies. Bonora et al [16] reported an incidence rate of 9.1 and 11.9 for 1000 person-years in people aged 50–59 and 60–69 years respectively, with no difference between gender. Bruno et al reported incidence rates of 1.5 and 0.76 for 1000 person-years respectively in men and women aged 45–49 years. We found incidence rates of 6.7 and 3.5 respectively for males and females aged 45–54 years and incidence rates of 12.8 and 7.6 for 1000 person-years respectively for males and females aged 55–64 years.

Fleming et al [18] reported a prevalence of diabetes in eight European countries for people aged 45–64 years ranging from 33.3 to 58.1 per 1000 and from 21 to 53 per 1000 in males and females respectively. We found at the start of our study a prevalence of 40.1 per 1000 in men aged 45–54 years, 91.2 per 1000 in men aged 55–64 years, 21 per 1000 in women aged 45–54 years and 55 per 1000 in women aged 55–64 years.

The results about hospitalisation for all causes and for cardiovascular and renal diseases is consistent with the expected burden of major complications in diabetic people and with previous reports [22]. For example, Bo et al in a recent paper [10] reported standardised hospital admission ratios for heart diseases in diabetic subjects aged 30–64 years of 230% for males and 341% for females: even if not directly comparable for several reasons (different classes of age, different aggregations of clinical conditions, different measures of association) our findings about this issue are very similar in a qualitative and a quantitative point of view (relative risk of hospitalisation for acute myocardial infarction in diabetic subjects aged 45–64 years of 3.2 and 6.2 for males and females respectively). An important and relatively novel finding of this study is that diabetes has a great impact on occurrence of cardiovascular diseases in people with otherwise low risk, and confirm the disease partially abolish the difference in the burden of cardiovascular diseases between genders: the DAI study assessed the prevalence of cardiovascular complications in diabetic subjects and showed that diabetic females present a prevalence of cardiovascular complication as high as diabetic males or even higher [20]. Donnan et al [21] report a relative risk of hospitalization for many causes in diabetic subjects respect to non diabetic people as a single estimate adjusted for age and sex. We prefer show age and sex specific estimates because in this manner we can describe the particularly great impact of diabetes in younger subjects and in females.

The relationship between diabetes and mortality identifies some features common to hospitalisation: absolute risk greater in males and older people and relative risk greater in females and younger subjects. In elderly diabetic subjects the mortality rates were similar to non diabetic subjects, with relative risk similar between genders: this means that the effect of diabetes on mortality tends to faint when age increases. The association between diabetes and mortality, that substantially disappear with the increase of age, is coherent with other studies [7].

Although in this study we limit our analysis to few items, other aspects should be investigated with a similar approach such as the follow-up of a cohort of incident cases with the evaluation of the evolution in antidiabetic treatment, the prevalence of other cardiovascular risk factors assessed by pharmacological treatment.

The present study has some limitations. We can trace only subjects with known diabetes: i.e. subjects who are utilizing health services. Moreover, subjects with known diabetes could escape to identification with current data if he/she doesn't assumes antidiabetic drugs nor requires the certificate for exemption from medical charges (for example all subjects aged 65 years or more are free from the great part of the out-of-hospital medical assistance). However, these limitations should not underestimate the occurrence of diabetes to a significant extent because diabetic subjects who did not assume antidiabetic drugs and not traceable in the EMC database are a little part of the diabetic population.

Another limit of this study is the lack of validation. However, we believe that this is a minor limitation since the high sensitivity of antidiabetic drugs in identifying diabetic people has been established by several studies in different settings. Because of the undercoding of diabetes in hospital discharge records, we assessed the hospitalisations of diabetic patients with record-linkage between the HDRs archive and the diabetic population otherwise identified. We defined the causes of hospitalisation by preferentially using codes with established accuracy (acute myocardial infarction [23], stroke [24]), or, otherwise widely used in scientific literature or by public agencies [25]. Because of the low accuracy of the diagnosis of diabetes in death certificates we refer to these only for the assessment of the death. Our data are consistent with other published studies on the same epidemiological aspects carried out with other more accurate but more expensive methodologies.

We did not distinguish between type 1 and type 2 diabetes mellitus: we did so because with current data a clear distinction is not possible. We can classify diabetic subjects according to treatment or to age of diagnosis; unfortunately, we lack data on the presence of ICA or GADA auto-antibodies or data about the levels of C-peptide. We are aware that the excess of cardiovascular and renal complications in diabetic subjects is due not only to diabetes but even (mainly) to other risk factors: hypertension, dyslipidemia, obesity (the metabolic syndrome); however we outline two important elements: first, hyperglycaemia per se even under the diabetic range may lead to the development of cardiac diseases [26]; second, the approach to the diabetic patients must consider these subjects as a whole, with careful attention to the control of all cardiovascular risk factor (in this light we can see diabetes as an indicator of high cardiovascular risk).

## Conclusion

The main strength of this study is the ability to give considerable informations about the prevalence and incidence of diabetes and its complications in a manner suitable to policy-makers for health planning: sufficient accuracy, timely availability, low cost and high sustainability. Another important aspect is the finding that diabetes has a major effect for the risk of cardiovascular diseases in people otherwise at low risk.

## Abbreviations

HDR: Hospital Discharge Record

MD: Mortality Data

DP: Drug Prescriptin

EMC: Exemption of Medical Charge

ICD-9-CM: International Classification of Diseases – ninth version – Clinical Modification

ICD-9: International Classification of Diseases – ninth version

ATC: Anatomical Therapeutic Chemical classification system

HbA1C: glycated haemoglobin A1C

ECG: electrocardiogram

## Authors' contributions

SB conceived of the study, participated in its design, planned the statistical analysis, drafted the manuscript

CV performed the statistical analysis

UF participated in the study design and drafted the manuscript

ES performed the statistical analysis

FA planned, performed and revised the statistical analysis

AA helped to draft and revised the manuscript

MA participated in the study design and collected the data

PS conceived of the study and participated in its design

All authors read and approved the final manuscript
